# Looking at Extremes without Going to Extremes: A New Self-Exciting Probability Model for Extreme Losses in Financial Markets

**DOI:** 10.3390/e22070789

**Published:** 2020-07-20

**Authors:** Katarzyna Bień-Barkowska

**Affiliations:** Institute of Econometrics, Warsaw School of Economics, Madalińskiego 6/8, 02-513 Warsaw, Poland; katarzyna.bien@sgh.waw.pl

**Keywords:** forecasting market risk, value at risk, extreme returns, peaks over threshold, self-exciting point process, discrete-time models, generalized Pareto distribution

## Abstract

Forecasting market risk lies at the core of modern empirical finance. We propose a new self-exciting probability peaks-over-threshold (SEP-POT) model for forecasting the extreme loss probability and the value at risk. The model draws from the point-process approach to the POT methodology but is built under a discrete-time framework. Thus, time is treated as an integer value and the days of extreme loss could occur upon a sequence of indivisible time units. The SEP-POT model can capture the self-exciting nature of extreme event arrival, and hence, the strong clustering of large drops in financial prices. The triggering effect of recent events on the probability of extreme losses is specified using a discrete weighting function based on the at-zero-truncated Negative Binomial (NegBin) distribution. The serial correlation in the magnitudes of extreme losses is also taken into consideration using the generalized Pareto distribution enriched with the time-varying scale parameter. In this way, recent events affect the size of extreme losses more than distant events. The accuracy of SEP-POT value at risk (VaR) forecasts is backtested on seven stock indexes and three currency pairs and is compared with existing well-recognized methods. The results remain in favor of our model, showing that it constitutes a real alternative for forecasting extreme quantiles of financial returns.

## 1. Introduction

Forecasting extreme losses is at the forefront of quantitative management of market risk. More and more statistical methods are being released with the objective of adequately monitoring and predicting large downturns in financial markets, which is a safeguard against severe price swings and helps to manage regulatory capital requirements. We aim to contribute to this strand of research by proposing a new self-exciting probability peaks-over-threshold (SEP-POT) model with the prerogative of being adequately tailored to the dynamics of real-world extreme events in financial markets. Our model can capture the strong clustering phenomenon and the discreteness of times between the days of extreme events.

Market risk models that account for catastrophic movements in security prices are the focal point in the practice of risk management, which can clearly be demonstrated by repetitive downturns in financial markets. The truth of this statement cannot be more convincing nowadays as global equity markets have very recently reacted to the COVID-19 pandemic with a plunge in prices and extreme volatility. The coronavirus fear resulted in panic sell-outs of equities and the U.S. S&P 500 index plummeted 9.5% on 12 March 2020, experiencing its worst loss since the famous Black Monday crash in 1987. Directly 2, 4, 6, and 7 business days later, the S&P 500 index registered additional huge price drops amounting to, correspondingly, 12%, 5.2%, 4.3%, and 2.9%, respectively. At the same time, the toll that the COVID-19 pandemic took on European markets was also unprecedented. For example, the German bluechip index DAX 30 plunged 12.2% on 12 March 2020, which was followed by a further 5.3%, 5.6%, 2.1% losses, correspondingly, 2, 4, and 7 business days later. The COVID-19 aftermath is a real example that highlights the strong clustering property of extreme losses.

One of the most well-recognized and widely used measures of exposure to market risk is the value at risk (VaR). VaR summarizes the quantile of the gains and losses distribution and can be intuitively understood as the worst expected loss over a given investment horizon at a given level of confidence [1]. VaR can be derived as a quantile of an unconditional distribution of financial returns, but it is much more advisable to model VaR as the conditional quantile, so that it can capture the strongly time-varying nature of volatility inherent to financial markets. The volatility clustering phenomenon provides the reason for using the generalized autoregressive conditional heteroskedasticity (GARCH) models to derive the conditional VaR measure [2]. However, over the last decade, the conventional VaR models have been subject to massive criticism, as they failed to predict huge repetitive losses that devastated financial institutions during the global crisis of 2007–2008. Therefore, special focus and emphasis is now placed on adequate modeling of extreme quantiles for the conditional distribution of financial returns rather than the distribution itself.

One of the relatively recent and intensively explored approaches to modeling extreme price movements is a dynamic version of the POT model which relies on the concept of the marked self-exciting point process. Unlike the GARCH-family models, POT-family models do not act on the entire conditional distribution of financial returns. Instead, their focus moves to the distribution tails where—in order to account for their heaviness—the probability mass is usually approximated with the generalized Pareto distribution. Early POT models described the occurrence of extreme returns as realizations of an independent and identically distributed (i.i.d.) variable, which led to VaR estimates in the form of unconditional quantiles. One of the first dynamic specifications of POT models that took into account the volatility clustering phenomenon and allowed economists to perceive VaR as a conditional quantile was a two-stage method developed in [3]. This method required estimating an appropriately specified GARCH-family model in the first stage and fitting the POT model to GARCH residuals. A new avenue for forecasting VaR was opened up when the point-process approach to POT models was released in [4]. This methodology was later extended in several publications [5,6,7,8,9,10,11,12,13,14]. The benefit of this model is that it does not require prefiltering returns using GARCH-family estimates while at the same time it can capture the clustering effects of extreme losses and maintain the merits of the extreme value theory. The point-process POT model approximates the time-varying conditional probability of an extreme loss over a given day with the help of a conditional intensity function that characterizes the arrival rate of such extreme events. The intensity function can either be formulated in the spirit of the self-exciting Hawkes process [4,5,10,11,12] (which is extensively used in geophysics and seismology), in the form of the observation-driven autoregressive conditional intensity (ACI) model [13], or using the autoregressive conditional duration (ACD) models [6,7,8] (the last two methodologies were very popular in the area of market microstructure and the modeling of financial ultra-high-frequency data [15,16,17]). In all cases, the timing of extreme losses depends on the timing of extreme losses observed in the past.

**Figure 1:**
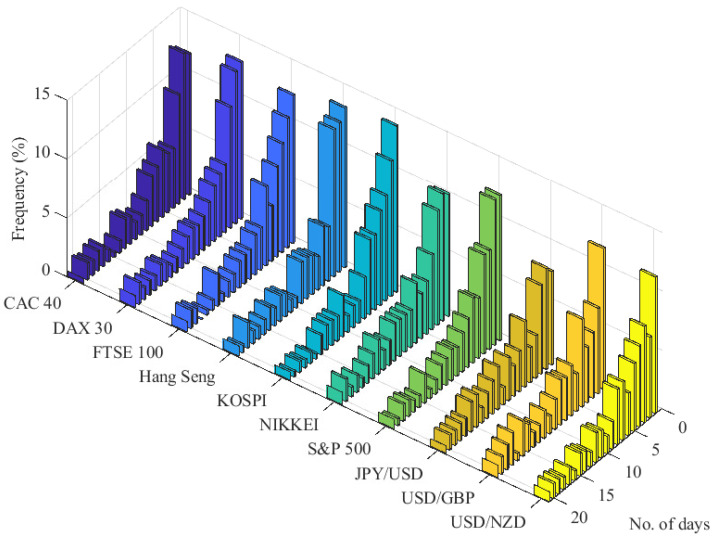
Frequency histogram for the time intervals (in number of days) between subsequent extreme losses for seven equity indexes and three FX rates between January 1981 and March 2020.

This study does not strictly rely on the above mentioned point process approach to POT models. The discrete-time framework of our SEP-POT model is motivated by observation of real-world financial data measured daily, which is the most common frequency used in POT models of risk. The empirical analysis put forward in this paper is based on the daily log returns of seven international stock indexes (i.e., CAC 40 (France), DAX 30 (Germany), FTSE 100 (United Kingdom), Hang Seng (Hong Kong), KOSPI (Korea), NIKKEI (Japan), and S&P 500 (U.S.)) as well as the daily log returns of three currency pairs (JPY/USD, USD/GBP, USD/NZD). The daily log returns for the equity market were calculated from the adjusted daily closing prices downloaded from the Refinitiv Datastream database. The foreign exchange (FX) rates were obtained from the Federal Reserve Economic Data repository and are measured in following units: Japanese Yen to one U.S. Dollar (JPY/USD), U.S. Dollars to one British Pound (USD/GBP), U.S. Dollars to one New Zealand Dollar (USD/NZD). Extreme losses are defined as the daily negated log returns (log returns pre-multiplied by −1) whose magnitudes (in absolute terms) are larger than a sufficiently large threshold, *u*. Figure 1 shows that for *u* corresponding to the 0.95-quantile of the unconditional distribution of negated log returns, the daily measurement frequency, and the broad set of financial instruments, the relative frequency mass of the time interval between subsequent extreme losses is concentrated on small integer values. Indeed, about 45% of all such durations is distributed on distinct discrete values of 1–5 days, and the most frequent time span between subsequent extreme losses is one day (about 12–13% of cases).

The SEP-POT model relates to the published work on the point-process approach to POT models but is consistent with the observed discreteness of threshold exceedance durations. Thus, in our model, the values of the time variable are treated as indivisible time units upon which extreme losses can be observed. As a result that the extreme losses are clustered, the model incorporates the self-exciting component. Accordingly, the extreme loss probability is affected by the series of time spans (in number of days) that have elapsed since all past extreme loss events. We apply the weighting function in the form of the at-zero-truncated Negative Binomial (NegBin) distribution that allows the influence of previous extreme losses to decay over time. The functional form of the extreme loss probability in our SEP-POT model is drawn from [18], where a very similar specification was proposed to depict the self-exciting nature of terrorist attacks in Indonesia and forecasted the probability of future terrorist attacks as a function of attacks observed in the past. Inspired by this work, we check the adequacy of such a discrete-time approach in the framework of POT models of risk. To this end, we perform an extensive validation of the SEP-POT model both in and out of sample and compare it with three widely-recognized VaR measures: one based on the self-exciting intensity (Hawkes) POT model, one derived from the exponential GARCH model with skewed Student’s t distribution (skewed-t-EGARCH) model, and the last one was delivered by the Gaussian GARCH model. The results for VaR at high confidence levels (>99%) remain in favor of the SEP-POT model, and hence, the model constitutes a real alternative for measuring the risk of large losses.

Section 2 outlines the point process approach to POT models, introduces the SEP-POT model, and outlines the backtesting methods used for model validation. Section 3 presents the empirical findings and discusses the extensive backtesting results. Finally, Section 4 concludes the paper and proposes areas for future research.

## 2. Methods

### 2.1. Self-Exciting Intensity POT Model

Consider $\left\{Y_{t}\right\}$ ($Y_{t}\in R$) denoting the stochastic process that characterizes the evolution of negated daily log returns on a financial asset, being the daily log returns pre-multiplied by −1. The convention of using negated log returns legitimizes treating extreme losses as observations that fall into the right tail of distribution. More precisely, the extreme losses are defined as such positive realizations of $Y_{t}$ that are larger than a sufficiently large threshold *u*. The magnitudes of extreme losses over a threshold *u*, (i.e., ${\bar{Y}}_{t}=Y_{t}-u$) will be referred to as the threshold exceedances. The time intervals between subsequent threshold exceedances will be referred to as threshold exceedance durations.

Let ${\left\{t_{i},Y_{t_{i}}\right\}}_{i\in \{1,2,\ldots ,n\}}$ denote an observed sample path of (1) the times when extreme losses are observed (i.e., $0<t_{i}<t_{i+1}$) and (2) the corresponding magnitudes of such losses (i.e., $Y_{t_{i}}$). If one pursued a continuous-time approach (i.e., assuming $t\in R_{+}$), the realized sequence ${\left\{t_{i},Y_{t_{i}}\right\}}_{i\in \{1,2,\ldots ,n\}}$ of extreme returns with their locations in time can be treated as an observed trajectory of the marked point process. Treating these instances of threshold exceedance as realizations of a random variable allows us to model the occurrence rate of extreme losses $Y_{t_{i}}$ at different time points $\left\{t_{i}\right\}$, for example, days. An excellent introduction to the theory and statistical properties of point processes can be found in [19].

The crucial concept in the point process theory is the conditional intensity function that characterizes the time structure of event locations, and hence, the evolution of the point process. The conditional intensity function is defined as follows:

(1) $$\begin{array}{ccc} \lambda (t|F_{t}) & = & \lim_{\Delta ↓0}\frac{\mathrm{Pr}\left[\left(N(t,t+\Delta ]\right)>0|F_{t}\right]}{\Delta }, \end{array}$$

where N(t,s] denotes a number of events in (t,s]. Note that the conditional intensity function can intuitively be treated as the instantaneous conditional probability of an event (per unit of time) immediately after time *t*. To account for the clustering of extreme losses, $\lambda (t|F_{t})$ depends on $F_{t}$ being an information set available at *t*, consisting of the complete history of event time locations and their marks, (i.e., $F_{t}\equiv \sigma \left\{\left(t_{i},Y_{t_{i}}\right),\forall i:t_{i}\leq t\right\}$). If $\lambda (t|F_{t})$ was constant over time (i.e., $\lambda (t|F_{t})=\lambda$) then for $t_{i}\in \left[0,\infty \right)$ the point process would correspond to a homogeneous Poisson point process with an arrival rate λ.

The notion of the conditional intensity facilitates the derivation of the conditional VaR measure. The VaR at a confidence level 1−q, (i.e., q∈(0,1) denotes a VaR coverage level), represents a *q*th quantile in the conditional distribution of financial returns. After taking advantage of working with the negated log returns and based on the notation introduced so far, the VaR (for a coverage level *q*) estimated for a day t+1 can be derived from the following equation:

(2) $$\begin{array}{c} \mathrm{Pr}(Y_{t+1}>y_{q,t+1}|F_{t})=q. \end{array}$$

Hence, the VaR for a coverage rate *q* is equal to $y_{q,t+1}$, because the probability that a (negated) return exceeds the threshold value $y_{q,t+1}$ over a day t+1 is equal to *q*. This probability can be further rewritten as a product of: (1) the probability of an extreme loss arrival (i.e., a threshold exceedance) over day t+1 (given $F_{t}$), and (2) the conditional probability that the size of this extreme loss is larger than $y_{q,t+1}$ (given that an extreme loss was observed over day t+1):

(3) $$\begin{array}{c} \mathrm{Pr}\left(Y_{t+1}>u|F_{t}\right)\mathrm{Pr}\left(Y_{t+1}>y_{q,t+1}|Y_{t+1}>u;F_{t}\right)=q. \end{array}$$

The early, classical POT model of the extreme value theory (EVT) (The EVT offers two major classes of models for extreme events in finance: (1) the block maxima method, which uses the largest observations from samples of i.i.d. data, and (2) the POT method, which is more efficient for practical application because it uses all large realizations of variables, provided that they exceed a sufficiently high threshold. A detailed exposition of these methods can be found in [20].) assumes that the financial return data is i.i.d. Hence, threshold exceedances are also i.i.d homogeneous Poisson distributed in time. Accordingly, the probability of observing a threshold exceedance over given day *t* is constant and can be estimated as a proportion of threshold exceedances in the sample (i.e., n/T, where *n* is the number of threshold exceedances and *T* denotes the length of time series of financial returns). By this logic, the standard POT model neglects repeated episodes of increased volatility and therefore also ignores the clustering property of extreme losses. As noted in [20], the standard POT model is not directly applicable to financial return data.

The more recent dynamic versions of the classical POT model found in several studies (i.e., [4,5,6,7,8,9,10,11,12,13,14]), are directly motivated by the behavior of the non-homogeneous Poisson point process, where the intensity rate of threshold exceedances, $\lambda (t|F_{t})$, can vary over time due to the temporal bursts in volatility. According to such a point process approach to POT models, the first factor on the left-hand side of Equation (3) (i.e., the conditional probability of a threshold exceedance over day t+1) can be derived based on the (time varying) conditional intensity function as follows:

(4) $$\begin{array}{cc} \mathrm{Pr}(Y_{t+1}>u|F_{t}) & =\mathrm{Pr}\left[N\left(t,t+1\right]>0|F_{t}\right]\\ & =1-\mathrm{Pr}\left[N\left(t,t+1\right]=0|F_{t}\right]\\ & =1-\exp\left(-\int_{t}^{t+1}\lambda \left(v|F_{v}\right)\;dv\right), \end{array}$$

because the probability of no events in (t,s] (i.e., N(t,s]=0) can be given as $\mathrm{Pr}\left(N\left(t,s\right]=0|F_{t}\right)=\exp\left(-\int_{t}^{s}\lambda \left(v|F_{v}\right)\;dv\right)$ [21].

The POT models use the Pickands–Balkema–de Haan’s theorem of EVT, which allows us to approximate the second factor on the left-hand side of Equation (3) (i.e., the conditional probability that $Y_{t+1}$ exceeds $y_{q,t+1}$, given that it surpassed the threshold *u*) using the generalized Pareto distribution, as follows:

(5) $$\begin{array}{cc} \mathrm{Pr}(Y_{t+1}-u>y_{q,t+1}-u|Y_{t+1}>u;F_{t}) & =1-\mathrm{Pr}(Y_{t+1}-u\leq y_{q,t+1}-u|Y_{t+1}>u;F_{t})\\ & \approx 1-F_{GP}\left(y_{q,t+1}-u|Y_{t+1}>u;F_{t}\right)\\ & ={\left(1+\xi \frac{y_{q,t+1}-u}{\sigma }\right)}_{+}^{-1/\xi }, \end{array}$$

where $F_{GP}\left(\cdot \right)$ denotes the cumulative distribution function of the generalized Pareto (GP) distribution with the scale parameter $\sigma \in R_{>0}$ and the shape parameter $\xi \in R_{\neq 0}$. If ξ→0, $F_{GP}\left(\cdot \right)$ tends to the cumulative distribution function of an exponential distribution.

Equations (3)–(5) provide the grounds for the derivation of $VaR_{q,t+1}$, as follows:

(6) $$\begin{array}{c} VaR_{q,t+1}=\left[{\left(\frac{q}{1-\exp\left(-\int_{t}^{t+1}\lambda \left(v|F_{v}\right)dv\right)}\right)}^{-\xi }-1\right]\cdot \frac{\sigma }{\xi }+u. \end{array}$$

The dynamic versions of the POT models benefit from both (1) the point process theory, which allows for the time-varying intensity rate of threshold exceedances, and hence, the clustering of extreme losses, and (2) the EVT, which allows us to account for the tail risk of financial instruments. Thus, the forecasts of daily VaR can be time-varying and react to the new information. (The early, classical POT models of EVT assume a constant intensity, λ, and a constant scale parameter of the GP distribution for threshold exceedances, σ. Accordingly, the VaR level is constant over time: $VaR_{q}=\left[{\left(\frac{qT}{n}\right)}^{-\xi }-1\right]\cdot \frac{\sigma }{\xi }+u$.) In empirical applications, appropriate dynamic specifications of selected components in Equation (6) are needed. One possible way of specifying the time-varying conditional intensity function $\lambda (t|F_{t})$ is provided by the Hawkes process [19]. The Hawkes process belongs to the class of so called self-exciting processes where past events can accelerate the occurrence of future events. Accordingly, the conditional intensity function is defined as follows:

(7) $$\begin{array}{cc} \lambda (t|F_{t-}) & =\mu +\int_{-\infty }^{t}w\left(t-v\right)\;dN\left(v\right)\\ & =\mu +\sum_{t_{i}<t}w\left(t-t_{i}\right), \end{array}$$

where $\mu \in R_{>0}$ denotes a constant and w(·) refers to a non-negative weighting function that captures the impact of past events, (i.e., extreme-loss days). Accordingly, each threshold exceedance at $t_{i}<t$ contributes an amount $w(t-t_{i})$ to the risk of an extreme loss at *t*. This is necessary to provide a convenient parametric functional form for w(·). The well-recognized weighting function that we apply in the empirical portion of this paper is an exponential kernel function, given as follows:

(8) $$\begin{array}{c} w(x)=\alpha \exp(-\beta \;x), \end{array}$$

where $\alpha \in R_{\geq 0}$, $\beta \in R_{\geq 0}$ are the parameters to be estimated. Accordingly, $\lambda (t|F_{t-})$ is based on the summation of exponential kernel functions evaluated at the time intervals that start at the times of previous extreme losses (i.e., $t_{i}$) and last up to time *t*. The parameters α and β capture, correspondingly, the scale (i.e., the amplitude) and the rate of decay characterizing an influence of past events on the current intensity. The point process features the self-excitation property because the conditional intensity function rises instantaneously after an extreme loss is registered, which, in the aftermath, triggers the arrival of next events. This mechanism results in the clustering effect, characterizing the location of extreme losses in time. The time-varying nature of the conditional intensity function results in the fluctuations of VaR (see Equation (6)). On top of the clustering feature, the self-exciting intensity POT (i.e., SEI-POT) model for VaR (c.f., Equation (6)) can be further extended to account for the serial correlation in the magnitudes of the threshold exceedances. This can be achieved by providing an appropriate dynamic model for the scale parameter of the GP distribution in Equation (5). In the empirical portion of this paper we use the following specification:

(9) $$\begin{array}{c} \sigma_{t}=\sigma \left({\bar{Y}}_{t}|F_{t-}\right)=\mu_{s}+\sum_{t_{i}<t}\alpha_{s}{\bar{Y}}_{t_{i}}\exp\left(-\beta_{s}\left(t-t_{i}\right)\right), \end{array}$$

where $\mu_{s}\in R_{>0}$, $\alpha_{s}\in R_{\geq 0}$, $\beta_{s}\in R_{\geq 0}$ denote the parameters to be estimated. Accordingly, the threshold exceedance magnitude is affected by the sizes and times of past threshold exceedances.

Unlike standard POT models, where the times of threshold exceedances are assumed to be i.i.d distributed according to the homogeneous Poisson process and the magnitudes of threshold exceedances are assumed to be i.i.d. GP distributed, the dynamic point-process-based variants of the POT models allow for a time-varying intensity rate of threshold exceedances and a time-varying expected magnitude of these threshold exceedances. Accordingly, the VaR is also time-varying. The interplay of fluctuations in $\lambda (t|F_{t})$ and in the scale parameter of the GP distribution for the threshold exceedances, $\sigma_{t}$, elevates VaR in turbulent periods of financial turmoil and decreases its level during calm periods. Hence, the VaR adjusts to observed market conditions.

### 2.2. Self-Exciting Probability POT Model

In this section we introduce the self-exciting probability POT model that obeys the natural distinction between the processes defined in discrete and continuous time. The structure of our model still draws from Equation (3), but we treat time as if it was composed of indivisible distinct units (days). Therefore, we refrain from approximating the conditional extreme loss probability using the conditional intensity function that characterizes the evolution of point process in continuous time. As a result that we formulate our model in discrete time, we directly describe the conditional probability of an extreme loss over day *t*, as follows:

(10) $$\begin{array}{ccc} p_{t} & = & \mathrm{Pr}\left(Y_{t}>u|F_{t-1}\right)=g\left({\tilde{\lambda }}_{t}\right), \end{array}$$

where g(·)∈(0,1) denotes a link function. One possible choice of specifying g(·) (cf., [18]) is:

(11) $$\begin{array}{c} p_{t}=1-\exp\left(-{\tilde{\lambda }}_{t}\right), \end{array}$$

where $p_{t}\in \left(0,1\right)$ if ${\tilde{\lambda }}_{t}>0$.

Based on [18], the conditional probability of an extreme loss arrival over day *t* can be described in a dynamic fashion that exposes the self-exciting nature of the SEP-POT model as follows:

(12) $$\begin{array}{c} {\tilde{\lambda }}_{t}=\mu +\alpha \sum_{t_{i}<t}g\left(t-t_{i}\right), \end{array}$$

where $\mu \in R_{>0}$ denotes a constant determining a baseline probability, $\alpha \in R_{\geq 0}$ determines the scale (amplitude) of the impact that the time location of the *i*th past extreme-loss event exerts on $p_{t}$, and g(·)≥0 denotes the weighting function (i.e., discrete kernel function) that makes the past extreme-loss events less impactful than the more recent events. We specify g(·) as the probability function of the at-zero-truncated negative binomial (NegBin) distribution.

A probability function of a NegBin distribution is:

(13) $$\begin{array}{c} f\left(x;\omega ,\kappa \right)=\frac{\Gamma (\kappa +x)}{\Gamma (\kappa )\Gamma (x+1)}{\left(\frac{\kappa }{\kappa +\omega }\right)}^{\kappa }{\left(\frac{\omega }{\omega +\kappa }\right)}^{x},\;\;\;x=0,1,2,\cdots , \end{array}$$

where $\omega \in R_{>0}$ and $\kappa \in R_{>0}$ are the parameters of the distribution and E(u)=ω and $Var\left(u\right)=\omega +\omega^{2}/\kappa$. For κ→∞, the NegBin distribution converges to a Poisson distribution. For κ=1, the geometric distribution is obtained.

The at-zero-truncated NegBin distribution was formerly used in high-frequency-finance for modeling the non-zero price changes of financial instruments [22,23]. The probability function of at-zero-truncated NegBin distribution is given as g(x;ω,κ)=f(x;ω,κ)/(1−f(0;ω,κ)) (for x=1,2,…), where $f\left(0;\omega ,\kappa \right)={\left(\kappa /\left(\kappa +\omega \right)\right)}^{\kappa }$:

(14) $$\begin{array}{c} g\left(x;\omega ,\kappa \right)=\frac{\Gamma (\kappa +x)}{\Gamma (\kappa )\Gamma (x+1)}{\left[{\left(\frac{\kappa +\omega }{\kappa }\right)}^{\kappa }-1\right]}^{-1}{\left(\frac{\omega }{\omega +\kappa }\right)}^{x},\;\;\;x=1,2,\cdots , \end{array}$$

**Figure 2:**
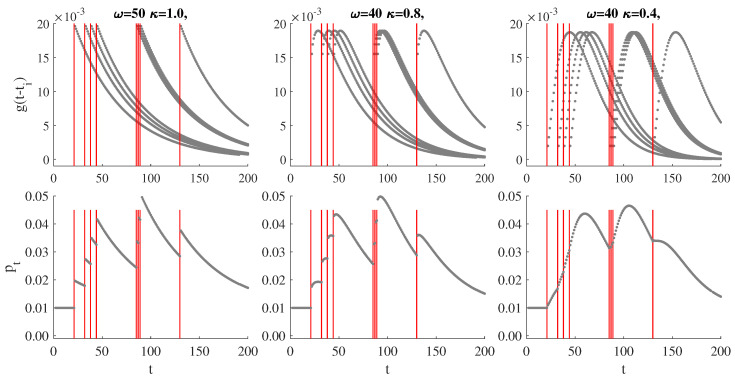
Illustration of the self-exciting probability model for eight events. Upper rows: Feasible shapes of the weighting functions $g(t-t_{i})$, $\forall i\;t_{i}<t$, at μ=0.01, α=0.5 (red lines indicate times of events). Lower row: The resulting probability $p_{t}$.

Figure 2 illustrates the self-exciting property of the SEP-POT model. The plots shown in the upper row depict the at-zero-truncated NegBin kernel functions evaluated at the time distances to previously observed events (i.e., $g\left(t-t_{i}\right)\;\forall i:\;t_{i}<t$). The impact of past events on $p_{t}$ diminishes with time and the shape of decay is determined by parameters ω and κ. The scale of this impact is determined by α. The resulting conditional probability function of an extreme loss arrival is therefore based on the summation of the weighted kernel functions based on all the backward recurrence times. The choice of an at-zero-truncated NegBin distribution guarantees flexibility in feasible shapes of the weighting function to properly reflect the dynamic properties of the data.

Like in existing dynamic extensions of the POT methodology, the threshold exceedance magnitudes in the SEP-POT model are described using the generalized Pareto distribution with the time-varying scale parameter. We specify this parameter as follows:

(15) $$\sigma_{t}=\sigma \left({\bar{Y}}_{t}|F_{t-1}\right)=\mu_{s}+\alpha_{s}\sum_{t_{i}<t}{\bar{Y}}_{t_{i}}g_{s}\left(t-t_{i};\omega_{s}\right),$$

where $\mu_{s}\in R_{>0}$ is a constant, $\alpha_{s}\in R_{\geq 0}$ is a scale parameter, and $g_{s}\left(x;\omega_{s}\right)$ (for x=1,2,…,) denotes the nonnegative discrete weighting (kernel) function. For this purpose, we use a PDF of a geometric distribution with parameter $\omega_{s}\in R_{>0}$, because it constitutes a natural discrete counterpart to an exponential distribution used in the continuous-time framework of the SEI-POT model (see Equation (9)). Hence, the magnitude of the threshold exceedance awaited at *t* is affected by the times and sizes of all previously observed threshold exceedances. The monotonically decaying weighting function allows distant events to affect the magnitudes of losses less than the recent events do.

The SEP-POT model assumes that the density function $f_{Y_{t}}^{u}\left(y_{t}|F_{t-1}\right)$, depicting the right tail of the distribution of the negated financial returns, has the following form:

(16) $$\begin{array}{ccc} f_{Y_{t}}^{u}\left(y_{t}|F_{t-1}\right) & = & p_{t}^{1_{\left\{t=t_{i}\right\}}}\cdot {\left(1-p_{t}\right)}^{\left(1-1_{\left\{t=t_{i}\right\}}\right)}\\ & \cdot & {\left(\frac{1}{\sigma_{t}}{\left(1+\xi \frac{y_{t}-u}{\sigma_{t}}\right)}_{+}^{-1/\xi -1}\right)}^{1_{\left\{t=t_{i}\right\}}}, \end{array}$$

which means that $Y_{t}$ either surpasses the threshold *u*, i.e., belongs to the right tail of distribution ($1_{\left\{t=t_{i}\right\}}=1$), and hence, is drawn from the generalized Pareto distribution with probability $p_{t}$, or does not belong to the distribution tail $\left(1_{\left\{t=t_{i}\right\}}=0\right)$ with probability $1-p_{t}$.

This reasoning allows us to formulate the log-likelihood function of the SEP-POT model as the sum of two log-likelihoods as follows:

(17) $$\begin{array}{c} \ln L=\ln L_{1}+\ln L_{2}, \end{array}$$

where:

(18) $$\begin{array}{ccc} \ln L_{1} & = & \sum_{t=1}^{T}\left[1_{\left\{t=t_{i}\right\}}\ln\left(p_{t}\right)+\left(1-1_{\left\{t=t_{i}\right\}}\right)\ln\left(1-p_{t}\right)\right]\\ & = & \sum_{t=1}^{T}\left[1_{\left\{t=t_{i}\right\}}\ln\left(\exp\left({\tilde{\lambda }}_{t}\right)-1\right)-{\tilde{\lambda }}_{t}\right], \end{array}$$

and

(19) $$\begin{array}{c} \ln L_{2}=-\left(1/\xi +1\right)\sum_{i=1}^{n}\ln\left(1+\xi \frac{y_{t_{i}}-u}{\sigma_{t}}\right)-\sum_{i=1}^{n}\ln\left(\sigma_{t}\right). \end{array}$$

The VaR for a coverage rate *q* forecasted for day *t* (based on the information up to and including day t−1) can be derived from the SEP-POT model as follows:

(20) $$\begin{array}{ccc} q & = & \mathrm{Pr}\left(Y_{t}>u|F_{t-1}\right)\mathrm{Pr}\left(Y_{t}>y_{q,t}|Y_{t}>u;F_{t-1}\right)\\ & = & p_{t}{\left(1+\xi \frac{y_{q,t}-u}{\sigma_{t}}\right)}^{-1/\xi }. \end{array}$$

Hence:

(21) $$\begin{array}{c} VaR_{q,t}=\left[{\left(\frac{q}{1-\exp(-{\tilde{\lambda }}_{t})}\right)}^{-\xi }-1\right]\cdot \frac{\sigma_{t}}{\xi }+u. \end{array}$$

The SEP-POT model provides the grounds not only to derive the VaR, but also the expected shortfall (ES). Unlike the VaR, the ES is a coherent risk measure. It represents the conditional expectation of loss given that the loss lies beyond the VaR [24]. Accordingly, the ES corresponding to a coverage rate *q*, forecasted for a day *t* based on the information set up to and including day t−1 is defined as following:

(22) $$\begin{array}{c} ES_{q,t}=E\left(Y_{t}|Y_{t}>VaR_{q,t};F_{t-1}\right). \end{array}$$

Equation (22) can be also rewritten as follows:

(23) $$\begin{array}{c} ES_{q,t}=VaR_{q,t}+E\left(Y_{t}-VaR_{q,t}|Y_{t}>VaR_{q,t};F_{t-1}\right). \end{array}$$

The ES can be derived based on the standard definition of the mean excess function for the GP distribution. For $u^{\prime }>u$, the mean excess function $e(u^{\prime })$ corresponding to the GP distribution (where σ>0, 0<ξ<1) is defined as:

(24) $$\begin{array}{c} e\left(u^{\prime }\right)=E\left(Y_{t}-u^{\prime }|Y_{t}>u^{\prime }\right)=\frac{\sigma +\xi (u^{\prime }-u)}{1-\xi }. \end{array}$$

Hence, the expected size of losses exceeding the threshold $u^{\prime }$ is a linear function of $u^{\prime }-u$. The ES forecasts from the SEP-POT model can be derived by applying the definition of $e(u^{\prime })$ to Equation (23) and by specifying the scale parameter of the GP distribution, σ, according to Equation (15). This leads to the formula for ES as follows:

(25) $$\begin{array}{ccc} ES_{q,t} & = & VaR_{q,t}+\frac{\sigma_{t}+\xi \left(VaR_{q,t}-u\right)}{1-\xi }\\ & = & \frac{VaR_{q,t}+\sigma_{t}-\xi u}{1-\xi }. \end{array}$$

### 2.3. Backtesting Methods

We use four backtesting procedures to assess the accuracy of the VaR delivered by the SEP-POT model. Each of these methods refer to the notion of a VaR exceedance or a VaR violation, being a binary indicator function, $I_{t}$, defined as follows:

$$\begin{array}{c} I_{t}=\left\{\begin{array}{c} 1,\;\;\mathrm{for}\;\;\;Y_{t}>VaR_{q,t}\\ 0,\;\;\mathrm{for}\;\;\;Y_{t}\leq VaR_{q,t}. \end{array}\right. \end{array}$$

The backtesting is based on the comparison of forecasted daily VaR numbers with observed daily returns over a given period. A VaR exceedance occurs when an actual loss is larger than the VaR predicted for that day. If the SEP-POT model were a true data generating process, than, ∀t$\mathrm{Pr}(I_{t}=1|F_{t-1})=q$, which implies that the VaR violations would be i.i.d.

#### 2.3.1. Unconditional Coverage Test

The first test that we consider is a widely used unconditional coverage (UC) test [25] where the null hypothesis states that the proportion of VaR exceedances according to a risk model (i.e., π) matches with the assumed coverage level for VaR (i.e., *q*): $H_{0}:\;\pi =q$. The UC test is formulated as a likelihood ratio test which compares two Bernoulli likelihood functions. Asymptotically, as the number of observations *T* goes to infinity, the test statistic is distributed as $\chi^{2}$ with one degree of freedom:

(26) $$LR_{UC}=-2\ln\left\{q^{T_{1}}{\left(1-q\right)}^{1-T_{1}}/\left[{\left(T_{1}/T\right)}^{T_{1}}{\left(1-T_{1}/T\right)}^{1-T_{1}}\right]\right\}\;∼\;\chi_{1}^{2},$$

where $T_{1}$ denotes the number of VaR violations in the sample of *T* returns.

#### 2.3.2. Conditional Coverage Test

The second test is the conditional coverage (CC) that not only verifies the correct coverage but also sheds light on the independence of VaR violations over time [26]. This test was established in such a way that it aims to reject the VaR models when a risk model produces either the incorrect proportions or the clusters of exceedances. To this end, the process of VaR violations is described by a first-order Markov model and the CC test is based on the estimated transition matrix, as follows:

(27) $$\begin{array}{c} \left[\begin{array}{cc} {\hat{\pi }}_{00} & {\hat{\pi }}_{01}\\ {\hat{\pi }}_{10} & {\hat{\pi }}_{11} \end{array}\right]=\left[\begin{array}{cc} T_{00}/\left(T_{00}+T_{01}\right) & T_{01}/\left(T_{00}+T_{01}\right)\\ T_{10}/\left(T_{10}+T_{11}\right) & T_{11}/\left(T_{10}+T_{11}\right), \end{array}\right] \end{array}$$

where $\pi_{00}$ and $\pi_{01}$ denote, correspondingly, the conditional probability of no VaR violation and a VaR violation (today), given that yesterday there was no VaR violation. Analogously, $\pi_{11}$ and $\pi_{10}$ denote, correspondingly, the conditional probability of a VaR violation and no VaR violation (today) directly after a VaR violation yesterday. As given in Equation (27), the elements of the transition matrix are estimated with the actual proportions of VaR violations, where $T_{ij}$, for i∈{0,1},j∈{0,1} is the number of (negated) returns with the indicator function $I_{t}$ equal to *j* directly following an indicator’s value *i*. The CC null hypothesis states that the conditional probability of a VaR violation directly after another VaR violation is the same as the conditional probability of a VaR violation after no violation and, at the same time, it is equal to the assumed coverage level for VaR (i.e., $H_{0}:\;\pi_{01}=\pi_{11}=q$). Asymptotically, as the number of observations *T* goes to infinity, the test statistic $LR_{CC}$ is distributed as a $\chi^{2}$ with two degrees of freedom:

(28) $$LR_{CC}=-2\ln\left\{q^{T_{1}}{\left(1-q\right)}^{1-T_{1}}/\left[{\left(1-{\hat{\pi }}_{01}\right)}^{T_{00}}\;{\hat{\pi }}_{01}^{T_{01}}\;{\left(1-{\hat{\pi }}_{11}\right)}^{T_{10}}\;{\hat{\pi }}_{11}^{T_{11}}\right]\right\}\;∼\;\chi_{2}^{2}$$

However, because the CC test is established on the Markov property of the violation process, it is sensitive to the dependence of order one only. Therefore, the CC test cannot be used to verify whether the current VaR exceedance depends on the sequence of states that preceded the last one.

#### 2.3.3. Dynamic Quantile Conditional Coverage Test

The next two backtesting methods shed more light on the higher-order autocorrelation in the process of VaR violations. They also allow us to conclude whether the violations are affected by some previously observed explanatory variables. The first test is a dynamic quantile (DQ) test [27] that is based on a hit function, as follows:

(29) $$Hit_{t}=I_{t}-q.$$

The correctly specified VaR model should form the $Hit_{t}$ sequence with a mean value insignificantly different from 0, because $Hit_{t}$ equals 1−q, each time $Y_{t}$ is larger than the daily VaR and −q, otherwise. Moreover, there should be no correlation between the current and the lagged values of the $Hit_{t}$ sequence or between the current values of the $Hit_{t}$ sequence and the current VaR. If the risk model corresponds to the true data generating process, the conditional expectation of $Hit_{t}$ should be 0 given any information known at t−1. The DQ test that we use in the empirical section of our paper can be derived as the Wald statistic from an auxiliary regression, as follows:

(30) $$Hit_{t}=\phi_{0}+\sum_{j=1}^{4}\phi_{j}Hit_{t-j}+\phi_{5}VaR_{q,t}+\varepsilon_{t}.$$

The null hypothesis states that the current value of a hit function (i.e., $Hit_{t}$) is not correlated with its four lags and the forecasted VaR (i.e., $VaR_{q,t}$ which is based on information known at t−1). Thus $H_{0}:\;\phi_{j}=0\;\forall j\in \left\{0,\ldots ,5\right\}$. Hence, the null hypothesis states that the coverage probability produced by a risk model is correct (i.e., $\phi_{0}=0$) and none of the five explanatory variables affects $Hit_{t}$. Hence, the DQ test statistic is asymptotically $\chi^{2}$ distributed with six degrees of freedom:

(31) $$DQ=\frac{{\mathrm{Hit}}^{\prime }X{\left(X^{\prime }X\right)}^{-1}X^{\prime }\mathrm{Hit}}{q(1-q)}\;∼\;\chi_{6}^{2},$$

where Hit denotes a T×1 vector with observations of $Hit_{t}$ variable and X denotes the standard T×6 matrix containing a column of ones and observations on the five explanatory variables at times t=1,…,T, according to the regression given in Equation (30).

#### 2.3.4. Dynamic Logit Conditional Coverage Test

The dynamic logit test of conditional coverage might be treated as an extension of the DQ conditional coverage test [28]. This method considers the dichotomous nature of VaR violations. Accordingly, instead of the linear regression given by Equation (30), this test is established based on the dynamic logit model for $I_{t}$: $E\left[I_{t}|F_{t-1}\right]=\mathrm{Pr}\left(I_{t}|F_{t-1}\right)=F\left(a_{t}\right)$, where F(·) denotes the cumulative distribution function of a logistic distribution and $a_{t}$ is specified as follows:

(32) $$a_{t}=\phi_{0}+\phi_{1}a_{t-1}+\phi_{2}I_{t-1}+\phi_{3}VaR_{q,t},$$

The autoregressive structure of Equation (32) allows us to better capture the dependence of a VaR violation probability upon possible explanatory factors. The null hypothesis is $H_{0}:\;\phi_{0}=F^{-1}\left(q\right),\;\phi_{1}=\phi_{2}=\phi_{3}=0$. Thus, the null states that the coverage probability delivered by a risk model corresponds to the assumed coverage rate for VaR (i.e., $\phi_{0}=F^{-1}\left(q\right)$) and none of regressors used in Equation (32) causes an incidence of VaR violation. The test statistic can be established as a likelihood ratio test statistic. Accordingly, it requires estimating the model given by Equation (32) and comparing its empirical log likelihood, $\ln L_{F}$, with the restricted log likelihood under the null $\ln L_{R}$. Under the null, the LR test statistic is $\chi^{2}$ distributed with four degrees of freedom:

(33) $$LR_{DL}=-2\left\{\ln L_{R}-\ln L_{F}\right\}\;∼\;\chi_{4}^{2}.$$

## 3. Results and Discussion

**Figure 3:**
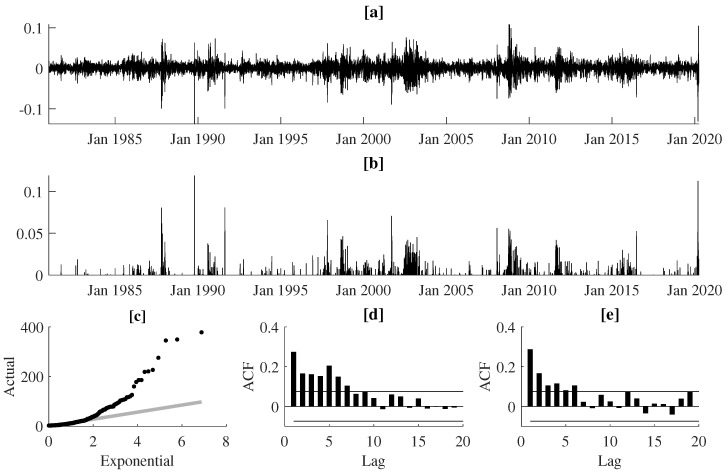
Panel (**a**) presents the daily log returns for the DAX index between Jan. 1981 and March 2020, panel (**b**) shows the corresponding ground-up threshold exceedances (i.e., the magnitudes of losses over the threshold *u*), panel (**c**) illustrates the quantile-quantile plot of inter-exceedance durations (in number of days) against the exponential distributions, and panels (**d**,**e**) present the autocorrelation functions for the inter-exceedance durations and the threshold exceedances, respectively.

In our empirical study we use daily log-returns from seven major stock indexes worldwide (CAC 40, DAX 30, FTSE 100, Hang Seng, KOSPI, NIKKEI, and S&P 500) and three currency pairs (JPY/USD, USD/GBP, USD/NZD). The CAC 40, DAX 30, and FTSE 100 are the major equity indexes in France, Germany, and U.K., respectively, and they are often perceived as the proxies or the real-time indicators for a much broader European stock market. The Hang Seng, KOSPI, and NIKKEI demonstrate the investment opportunity on the largest Asian equity markets in Hong Kong, South Korea, and Japan, respectively. S&P 500 constitutes a widely-investigated benchmark stock index reflecting the state of the overall U.S. economy. These seven indices monitor the state of the international equity market in its three global financial centers—western Europe, eastern Asia, and the U.S. As far as selection of the FX rates is concerned, according to [29], the JPY/USD and USD/GBP are the second and third most traded currency pair in the world, after EUR/USD (We did not investigate the EUR/USD currency pair due to a much smaller number of observations when comparing to the other time series; the euro was launched on 1 January 1999). The NZD/USD, often nicknamed as the Kiwi by FX traders, is a classical example of the commodity currency pair that co-fluctuates with the world prices of primary commodities (i.e., New Zealand exports oil, metals, dairy, and meat products). The New Zealand Dollar is also treated by international investors as a carry trade currency—therefore, it is very sensitive to interest rate risk. For each of these financial instruments we split the data spanning over a four-decades-long period into: (1) the in-sample data (i.e., 2 January 1981–31 December 2014) dedicated to the estimation and evaluation of our models and (2) the out-of-sample data (i.e., 2 January 2015–31 March 2020) which is reserved for VaR backtesting purposes. For each of the time series, the initial threshold *u* was set as the 95%-quantile of the in-sample unconditional distribution of negated log returns. Hence, the 5% largest negated returns were defined as extreme losses, which means that, on average, an extreme loss can be observed with probability 0.05. The selection of the threshold value *u* was a compromise between (1) the desired number of observations in the tail of the distribution to reduce noise and to ensure stability in parameter estimates (i.e., the lower the *u*, the more observations used for estimation) and (2) the goodness-of-approximation of the threshold exceedance distribution with the GP distribution (i.e., the higher the *u*, the better the approximation with the GP distribution). The latter issue was solved using two diagnostic tools, that confirmed the adequate goodness-of-fit of the conditional GP distribution. We used the D-test proposed in Ref. [30] and the $\chi^{2}$ test for uniformity of probability integral transforms (PIT) based on the GP density estimates. Figure 3 illustrates extreme losses corresponding to the German DAX 30 index between January 1981 and March 2020. The examination of panels [a] and [b] allows us to conclude that the periodic volatility bursts are paralleled with the strong clustering effects for both (1) the magnitudes of extreme losses and (2) the days that they occur. Indeed, the quantile-quantile (QQ) plot (panel [c]) comparing empirical quantiles of the time intervals between subsequent extreme-loss days against the quantiles of an exponential distribution proves that the times of extreme losses are not distributed according to the homogeneous Poisson point process. Clustering of extreme events is also demonstrated by the shape of the autocorrelation function (ACF), indicating significant positive autocorrelations in both time intervals between successive threshold exceedances and the observed magnitudes of such exceedances.

The descriptive statistics of the CAC 40, DAX 30, and FTSE 100 data are summarized in Table 1 (analogical results for the remaining time series can be obtained from the author upon request). We see that for the CAC 40, DAX 30, and FTSE 100, the threshold exceedances were obtained as the losses surpassing *u* that is equal to 0.021, 0.021, and 0.017, respectively. Out of 8574 (CAC 40), 8563 (DAX 30), and 7826 (FTSE 100) daily log returns in-sample, these threshold values allow us to expose, correspondingly, 429, 428, and 391 extreme losses that were used for the model estimation purposes. For the FTSE 100 index, we have less observations (corresponding to three years: 1981–1983), because the in-sample period starts on 3 January 1984, when the FTSE 100 index was established. Although the official base date for the DAX 30 index is 31 December 1987, the DAX 30 index was linked with the former DAX index which dates back to 1959. The official base date for the CAC 40 also begins on 31 December 1987, but between 2 January 1981 and 30 December 1987 it could be measured as the “Insee de la Bourse de Paris.” The threshold-exceedance durations cover a very wide range of observed values. For example, for the FTSE 100 index, the range spans from one day (with the relative frequency equal to 12.8% in-sample and 11.3% out-of-sample) up to 304 days in-sample or 205 days out-of-sample. In-sample, the largest threshold exceedance, equal to 0.114, was observed on the Black Monday of 20 October 1987 and it corresponded to a 12.22% decrease of the index. Out-of-sample, the maximum threshold exceedance is equal to 0.099 (a 10.87% plunge in the index) and was observed on the Black Thursday of 12 March 2020, being a single day in a chain of stock market crashes induced by the COVID-19 pandemic.

Realized gains and losses are measured over distinct days, and hence, the time spans between extreme losses are comprised of discrete time units (i.e., days). The scale of this phenomenon can be seen by looking at the considerable proportion of threshold exceedance durations equal to one, two, or three (business) days. Moreover, about 45% of such durations is less than or equal to five days and over 60% are less than or equal to ten days. Another striking observation from Table 1 is the clustering of extreme losses. Large losses tend to occur in waves, which is seen from the Ljung-Box test statistics Q(k) (where k∈{5,10,15}) for the lack of up to *k*th-order serial correlation. These test statistics are significantly different from zero, and hence, the null hypothesis of no autocorrelation in threshold exceedance durations must be rejected. Indeed, due to the COVID-19 outbreak, between 24 Februry and 31 March 2020 (i.e., over 27 business days) the CAC 40, DAX 30, and FTSE 100 suffered from as many as 10 (CAC40 and DAX 30) or 11 (FTSE 100) extreme losses (with the shortest and the longest threshold exceedance durations equal to one and five business days only, respectively). Extreme loss days tended to occur very close to each other, but this phenomenon is paralleled by the significant autocorrelation in the magnitudes of observed threshold exceedances. Based on the Ljung-Box test results, the null hypothesis of no autocorrelation in the threshold exceedance sizes needs to be rejected. The observed threshold exceedance durations are by their very nature discrete and feature strong positive autocorrelation. Therefore, our SEP-POT model is suitably tailored to this data.

The SEP-POT model was estimated by maximizing the log likelihood function given in Equations (17)–(19). To this end, we used the constrained maximum likelihood (CML) library of the Gauss mathematical and statistical system. The standard errors of the parameter estimates were derived from the asymptotic covariance matrix based on the (inverse) of a computed Hessian. Table 2 presents the estimation results for the CAC 40, DAX 30, and FTSE 100 (analogical results for the remaining time series can be obtained from the author upon request). The parameter estimates responsible for the self-excitement mechanism, both in the probability of threshold exceedances (i.e., $\hat{\alpha }$, $\hat{\omega }$, $\hat{\kappa }$) and the magnitudes of these exceedances (i.e., ${\hat{\alpha }}_{s}$, ${\hat{\omega }}_{s}$) are highly statistically significant. The parameter estimates for DAX 30 and CAC 40 indices look very much alike, especially for the conditional probability of threshold exceedances, which means that these two stock markets are closely related to each other.

**Figure 4:**
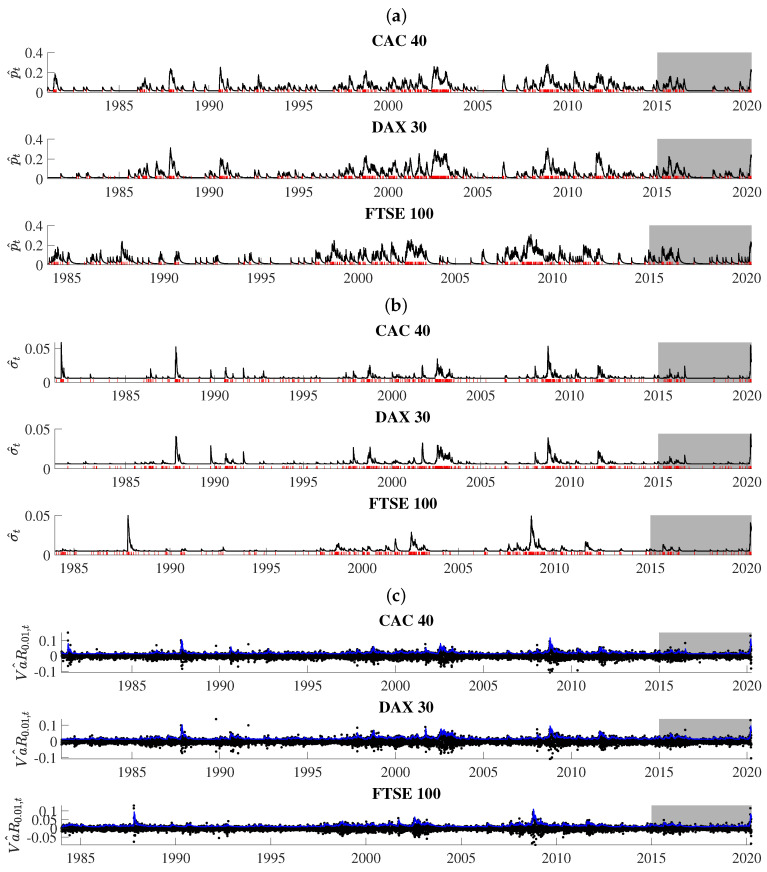
Estimation results from the SEP-POT models: the conditional probability of a threshold exceedance (i.e., $p_{t}$, panel (**a**)); the time-varying scale parameter of the generalized Pareto (GP) distribution for the magnitudes of threshold exceedances (i.e., $s_{t}$ panel (**b**)); the daily value at risk (VaR) at the confidence level 99 % (in blue color) that overlays the (negated) log returns (panel (**c**)). The days of extreme losses were marked in red. The shadowed area corresponds to the out-of-sample period.

Obtained series for ${\hat{p}}_{t}$, ${\hat{\sigma }}_{t}$, and ${V\hat{a}R}_{0.01,t}$ are illustrated in Figure 4. The extreme loss probability (i.e., ${\hat{p}}_{t}$) features a strong self-excitation property because it reacts to extreme-loss days with abrupt increases and, if there are no further intervening events, it slowly wanders in the downward direction. In calm and prosperous periods of the stock market history, the path of ${\hat{p}}_{t}$ rests on very low levels. However, in turbulent periods, when the location of extreme-loss days is very dense, ${\hat{p}}_{t}$ tends to involve very high numbers. More specifically, persistently elevated ${\hat{p}}_{t}$ levels can be seen during the market downturn of 2002–2003 and the global crisis of 2008–2009. For the CAC 40 and FTSE 100, the highest in-sample ${\hat{p}}_{t}$ level, equal to 0.2834 (CAC 40) and 0.3082 (FTSE 100), was reached on Monday, 24 November 2008. Both maximum values were triggered by a self-excitation mechanism during the prevailing stock market turmoil. Directly before 24 November 2008 the market suffered three consecutive extreme-loss days–November 19. (Wednesday), 20. (Thursday) and 21. (Friday). For the DAX 30 index, the in-sample ${\hat{p}}_{t}$ peaked to its highest level (0.3126) on 11 November 1987, in the aftermath of 10 steep losses that started on the Black Monday of 19 October. The last three were observed on three business days, 6–10 November 1987. Out-of-sample, the highest ${\hat{p}}_{t}$ levels of 0.2298 (CAC 40), 0.2416 (DAX 30), and 0.2339 (FTSE 100) corresponded to 24 March 2020 (CAC 40 and DAX 30) and 19 March 2020 (FTSE 100). COVID-19-induced anxiety before 24 March, resulted in the concentration of six threshold exceedances for CAC 40 and DAX 30 in March 2020 alone, where the last of these threshold exceedances took place just one day before the highest ${\hat{p}}_{t}$ level was reached on 23 March 2020.

Observed fluctuations of ${\hat{p}}_{t}$ are accompanied with the strongly time-varying behavior of ${\hat{\sigma }}_{t}$ (i.e., the estimate of the dispersion parameter in the conditional distribution of threshold exceedances). The losses exceeding *u* trigger upward jumps in both numbers, boosting the awaited probability and the size of a threshold exceedance. For the CAC 40 index, ${\hat{\sigma }}_{t}$ peaked to its highest level (0.059) on 15 May 1981, due to enormous panic and sell-offs on the Paris Bourse just days before Francois Mitterand announced hostile reforms for the stocks quoted at the Bourse. Indeed, the preceding days saw the CAC 30 index plunge by over 30%. The UK and German markets were mostly untouched by these French policy-oriented events, and the highest ${\hat{\sigma }}_{t}$ was registered on 27 October 1987 (FTSE 100) and 29 October 1987 (DAX 30) at the levels of 0.051 (FTSE 100) and 0.042 (DAX 30), just after a few huge price drops were observed including the famous Black Monday on 19 October 1987. Note, that the maximum ${\hat{\sigma }}_{t}$ levels do not have to coincide with those of ${\hat{p}}_{t}$. This is because ${\hat{\sigma }}_{t}$ is also affected by the magnitude of past threshold exceedances. For all data in this study, the highest out-of-sample ${\hat{\sigma }}_{t}$ levels were registered in the second half of March 2020.

The self-triggering nature of ${\hat{p}}_{t}$ and ${\hat{\sigma }}_{t}$ give rise to variations in daily VaR, as shown in the panel [c] of Figure 4. What catches special attention is that the obtained path of VaR estimates tends to adjust to both periods of calm and turmoil in the history of equity markets—it quickly reacts to price jumps and bursts in volatility and accounts for persistent swings in stock prices.

**Figure 5:**
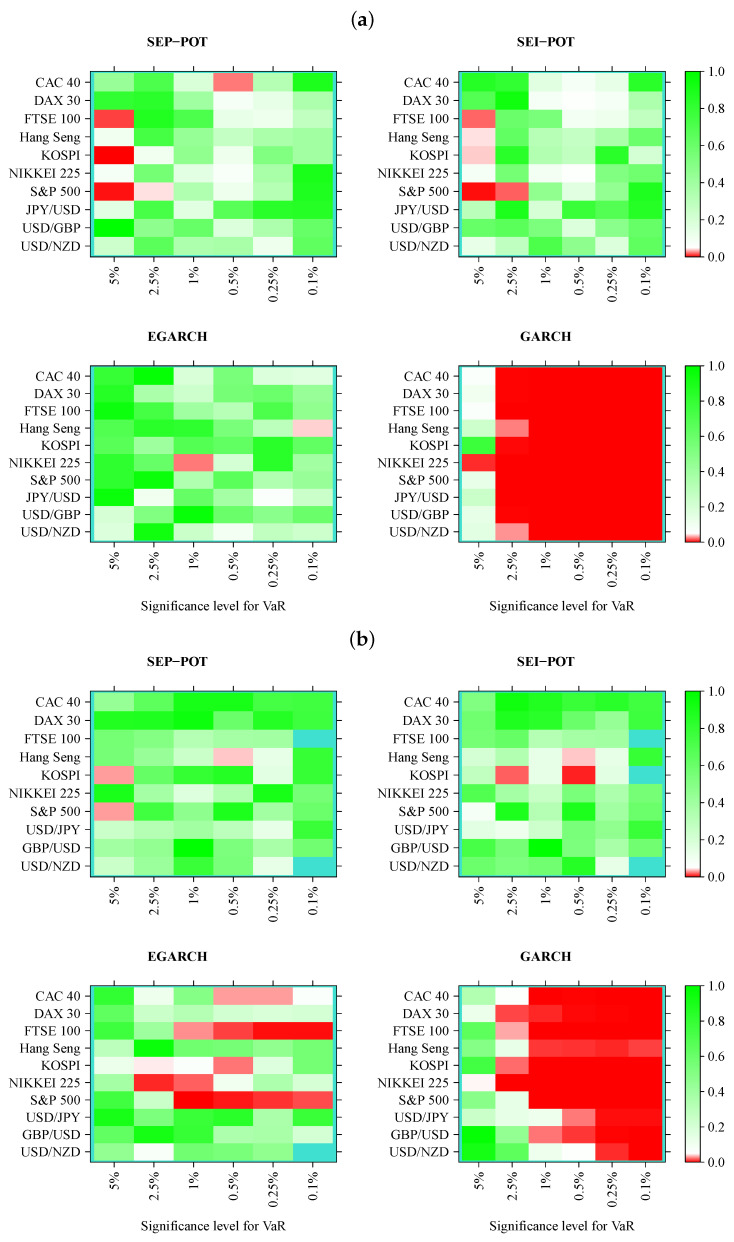
Heatmap charts showing *p*-value for the in-sample (panel (**a**)) and out-of-sample (panel (**b**)) for unconditional coverage (UC) tests. VaR series was calculated from the self-exciting probability POT model (SEP-POT), self-exciting intensity (Hawkes) POT model (SEI-POT), the EGARCH(1,1) model with the skewed-t distribution (EGARCH), and standard GARCH(1,1) model with normally-distributed innovations (GARCH). The squares of the heatmaps in the shades of red correspond to *p*-value < 0.05. The rectangles in turquoise color correspond to no VaR exceedances.

**Figure 6:**
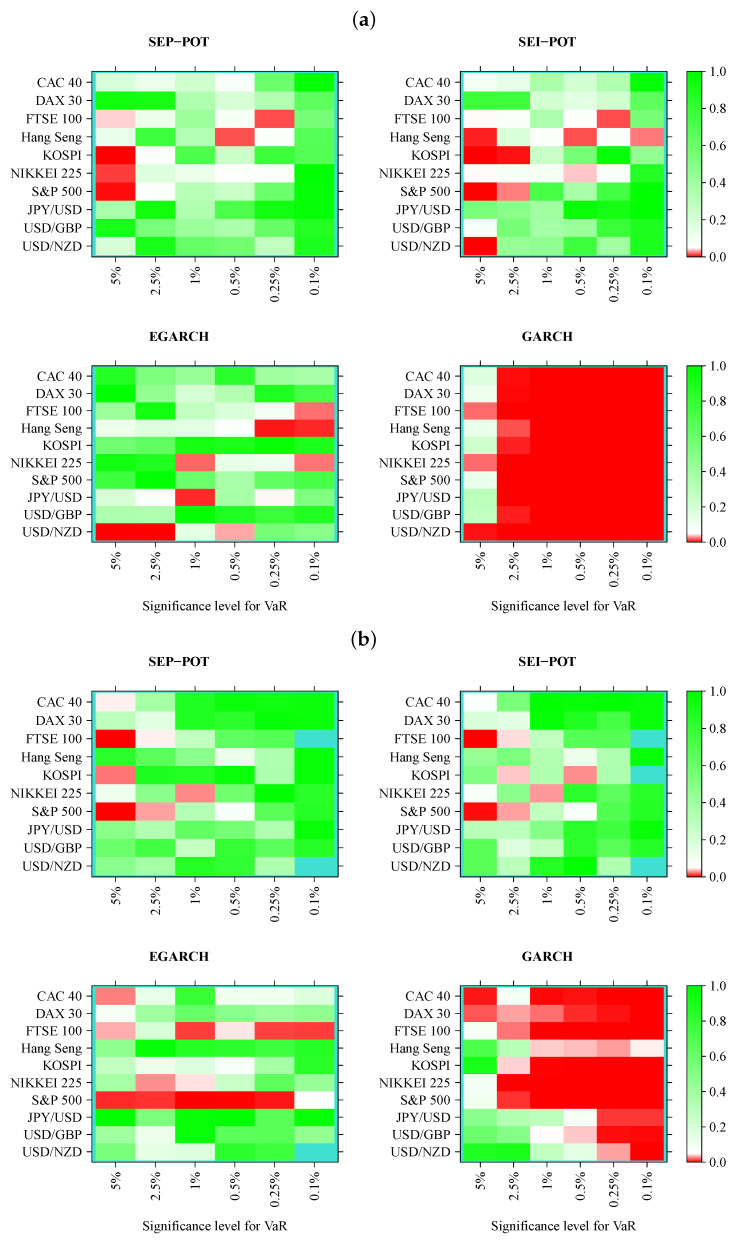
Heatmap charts showing *p*-value for the in-sample (panel (**a**)) and out-of-sample (panel (**b**)) for conditional coverage (CC) tests. VaR series was calculated from the self-exciting probability POT model (SEP-POT), self-exciting intensity (Hawkes) POT model (SEI-POT), the EGARCH(1,1) model with the skewed-t distribution (EGARCH), and standard GARCH(1,1) model with normally-distributed innovations (GARCH). The squares of the heatmaps in the shades of red correspond to *p*-value < 0.05. The rectangles in turquoise color correspond to no VaR exceedances.

**Figure 7:**
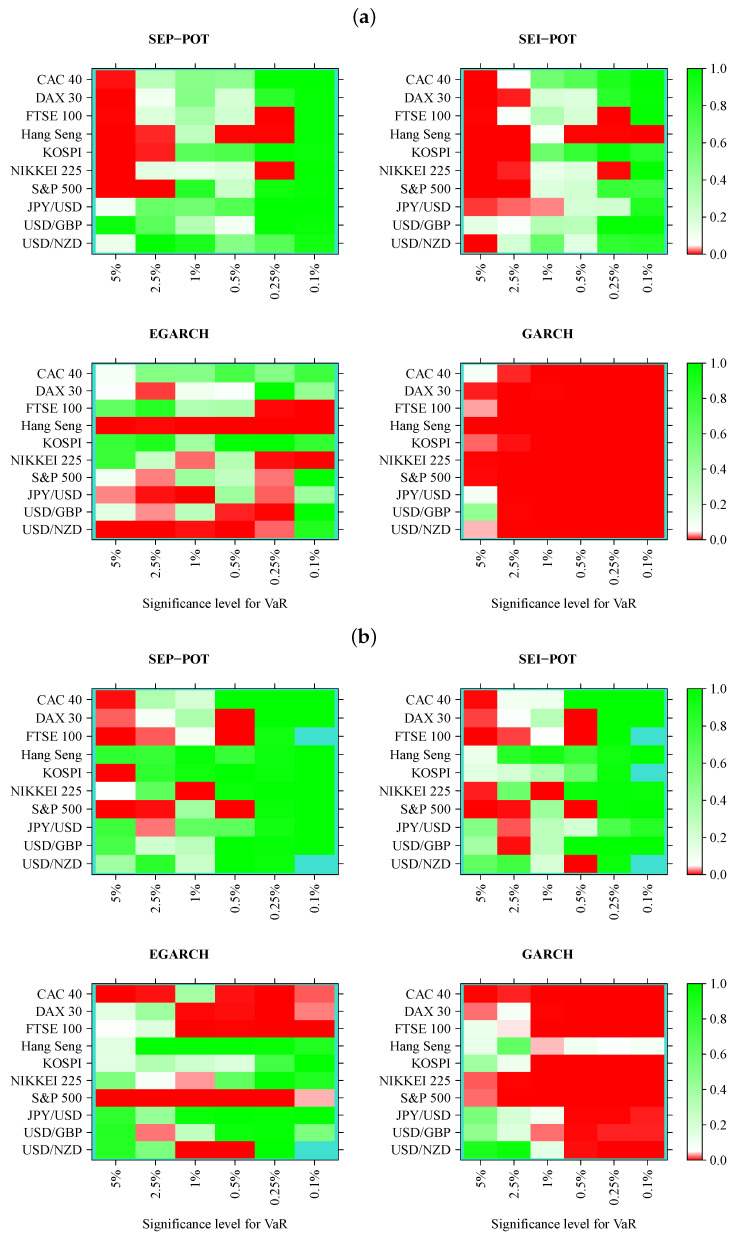
Heatmap charts showing *p*-value for the in-sample (panel (**a**)) and out-of-sample (panel (**b**)) for dynamic quantile (DQ) conditional coverage tests. VaR series was calculated from the self-exciting probability POT model (SEP-POT), self-exciting intensity (Hawkes) POT model (SEI-POT), the EGARCH(1,1) model with the skewed-t distribution (EGARCH), and standard GARCH(1,1) model with normally-distributed innovations (GARCH). The rectangles of the heatmaps in the shades of red correspond to *p*-value < 0.05. The rectangles in turquoise color correspond to no VaR exceedances.

**Figure 8:**
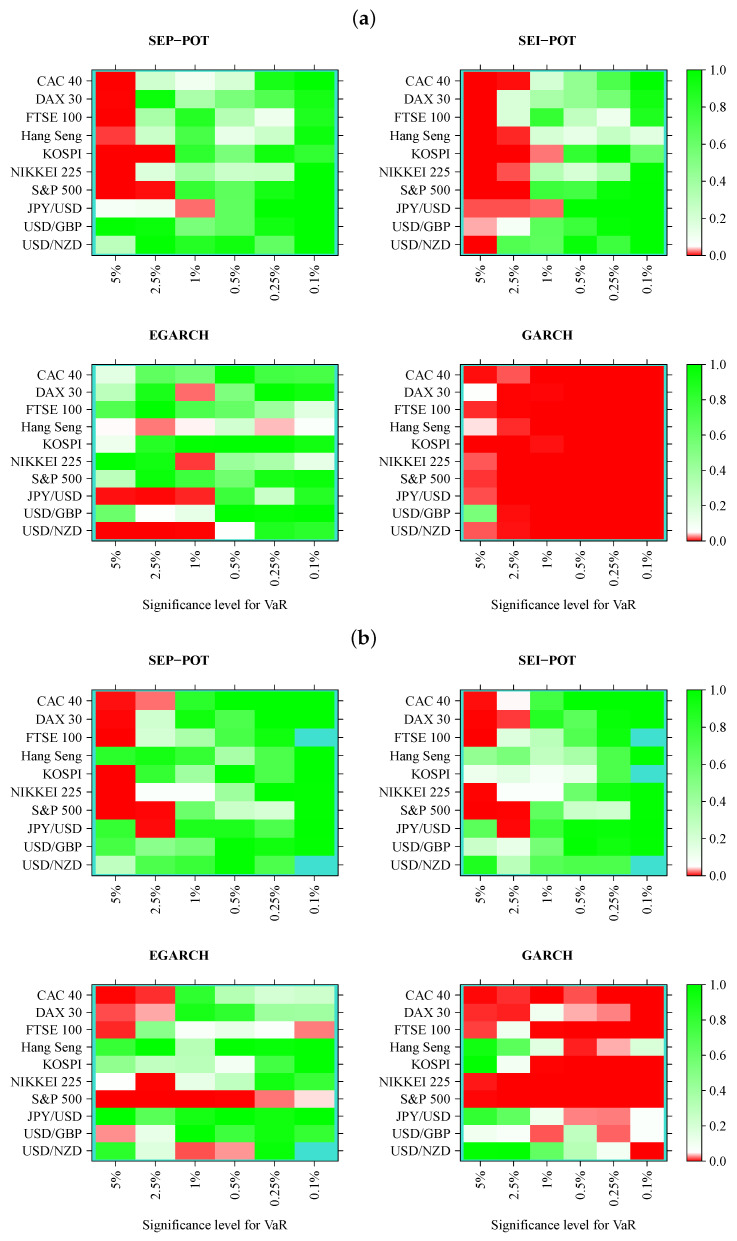
Heatmap charts showing *p*-value for the in-sample (panel (**a**)) and out-of-sample (panel (**b**)) for dynamic logit conditional coverage tests. VaR series was calculated from the self-exciting probability POT model (SEP-POT), self-exciting intensity (Hawkes) POT model (SEI-POT), the EGARCH(1,1) model with the skewed-t distribution (EGARCH), and standard GARCH(1,1) model with normally-distributed innovations (GARCH). The rectangles of the heatmaps in the shades of red correspond to *p*-value < 0.05. The rectangles in turquoise color correspond to no VaR exceedances.

We verified whether the SEP-POT model is appropriate for forecasting the daily VaR. To ensure a big-picture perspective over its usefulness in diverse practical applications, we derived the daily VaR levels for six assumed theoretical coverage rates (i.e., for q∈{0.05,0.025,0.01,0.005,0.0025,0.001}), and compared them with corresponding VaR numbers from three competing risk models (i.e., the self-exciting intensity (Hawkes) POT model (SEI-POT), the EGARCH(1,1) model with the skewed-t distributed innovations and the standard GARCH(1,1) model with normally-distributed innovations). For the sake of fair comparison between the four risk models under study, the accuracy of VaR forecasts was validated with four backtesting procedures. Moreover, each of these statistical routines was distinctly applied to examine the following: (1) the in-sample goodness-of-fit and (2) the out-of-sample accuracy. Considering ten financial instruments under study, six coverage levels for VaR (*q*) and four models (SEP-POT VaR, SEI-POT VaR, skewed-t-EGARCH VaR, and Gaussian GARCH VaR), we ended up with 240 VaR series in-sample and 240 series out-of-sample. Therefore, for clarity of exposition, the backtesting results were summarized in the form of heatmap graphs (cf., Figure 5, Figure 6, Figure 7 and Figure 8). Heatmaps use a grid of colored rectangles across two axes where the horizontal axis corresponds to the assumed VaR coverage level and the vertical axis corresponds to the financial instrument under study. The color of each little rectangle (in shades of red and green) reflects the *p*-value of a backtesting procedure. The white colour corresponds to a *p*-value equal to 0.05. The darker the red color indicates an increasingly smaller *p*-value, one that it is less than 0.05. The darker the tone of green indicates an increasingly higher *p*-value, one that it is larger than 0.05. For example, panel [a] of Figure 5 presents the *p*-values corresponding to the UC test statistics. Each of the four heatmaps in panel [a] refers to the VaR delivered from a different model: the SEP-POT, SEI-POT, skewed-t-EGARCH, and Gaussian GARCH.

According to the UC test results, the VaR based on the SEP-POT, SEI-POT, and skewed-t-EGARCH models produce, in-sample, a rather accurate proportion of violations. The best in-sample results were delivered by the skewed-t-EGARCH model; however, its superiority diminishes out-of-sample, where the skewed-t-EGARCH model failed in 13 out of 60 instances. Out-of-sample, the SEP-POT VaR and SEI-POT VaR models rejected the null of correct coverage only three times. The EGARCH model seems to produce good VaR forecasts for high coverage levels (i.e., q=0.05). For q<0.05, the EGARCH VaR model is left behind the SEI-POT VaR model and SEP-POT VaR model. As expected, the advantage of VaR models based on POT methodology is most visible for the extreme quantiles. As far as the Gaussian GARCH VaR model is concerned, its performance is dramatically worse than other risk models both in-sample and out-of-sample. The model produces incorrect VaR forecasts for small *q* (i.e., q≤0.025), which can be explained by insufficient probability mass in the tails of Gaussian distribution.

The results of the CC test checking both the correct coverage and the lack of dependence of order one in VaR violations seem to support the SEP-POT VaR model (cf., Figure 6). The poorest fit corresponds to the highest *q* levels (i.e., q=0.05) because in such cases, the null of proper specification had to be rejected both in-sample and out-of-sample for FTSE 100, KOSPI, NIKKIEI, and S&P 500. However, the SEP-POT VaR model seems to be slightly superior than the SEI-POT VaR model. In sample, only in six instances out of 60 did the SEP-POT VaR model fail. For the SEI-POT VaR model, the number of failures was 10 and for the skewed-t-EGARCH VaR model it was nine. As in the case of the UC test, the CC test results indicate that the Gaussian GARCH VaR model rendered the worst fit—the null was not rejected in only seven cases, mainly for the lowest quantiles (i.e., for q=0.05). Out-of-sample, the SEP-POT and the SEI-POT models deliver the similar quality of daily VaR forecasts and both win over GARCH-family models.

Turning our attention to Figure 7, which illustrates the results of the DQ test, the first striking observation is that a much larger area of all heatmaps is marked with shades of red when compared to the results of the CC tests. Indeed, the DQ test is more demanding than the CC test because checks not only whether a VaR violation today is uncorrelated with the fact of a VaR violation yesterday but it also checks whether VaR violations are affected by some covariates from a wider information set, where we used the current VaR and the Hit variable observations from one to four days ago (as in original work [27]). The superiority of the SEP-POT VaR model over its competitors is clearly visible. Although the SEP-POT VaR model has a clear tendency to mis-specify VaR at the highest *q* levels (i.e., q=0.05), the DQ test results for the SEI-POT VaR and the VaR based on the GARCH family models are inferior. In-sample, the DQ test rejected 14 SEP-POT VaR models, 21 SEI-POT VaR models, 26 skewed-t-EGARCH VaR and 57 (i.e., nearly all) Gaussian GARCH VaR models. Out-of-sample, the advantage of the SEP-POT VaR model over the SEI-POT VaR model is less vivid—the first model failed in 12 instances and the latter failed in 14.

Figure 8 illustrates the results of the dynamic logit CC test. We can observe a systematic pattern as far as the SEP-POT VaR and SEI-POT VaR models are concerned. The area marked in red concentrates on the left-hand side of the heatmaps both in and out-of-sample, which means that VaR is mis-specified if derived for high coverage rates (i.e., q=0.05). This deficit of POT VaR models is recouped by their accuracy at low *q* levels. Indeed, for q≤0.005 in-sample and for q≤0.01 out-of-sample, both POT models are not able to reject the null. The SEP-POT VaR model was still slightly more successful than the remaining risk models. In-sample, it failed only 10 times (mainly for q=0.05), whereas the SEI-POT VaR model failed 18 times, the skewed-t-GARCH model failed ten times, yet the Gaussian GARCH VaR model managed to pass this test only two times. Out-of-sample, both POT VaR models were equally correct. For the SEP-POT and SEI-POT VaR model, the null of correct conditional coverage was rejected nine times. The dynamic logit CC test rejected the skewed-t-EGARCH model in 16 and the Gaussian GARCH in majority of cases.

The practical implications of the SEP-POT model stem from its suitability to provide adequate VaR and ES predictions. The VaR forecasts can be used by financial institutions as internal control measures of market risk. The adequacy of risk models used by financial institutions is of utmost importance for the market regulator. Commercial banks have used VaR models for several years to calculate regulatory capital charges using the internal model-based approach of the Basel II regulatory framework. According to the more recent recommendations of the Basel Committee on Banking Supervision (BCBS), banks should use ES to ensure a more prudent capture of “tail risk” and capital adequacy during periods of significant stress in the financial markets [31]. This attitude remains in line with the core objective of the dynamic POT models (including the SEP-POT model), as they focus on the quantification of both the forecasted probability and the awaited size of huge losses, also producing the time-varying ES forecasts. The recent Basel III accord, comprising a set of regulations developed by the BCBS, further reinforces the role of bank units responsible for internal model validations. For more about the current regulatory framework of market risk management see [32]. Despite the recent shift from VaR to ES models in the calculation of capital requirements, ES forecasts remain highly sensitive to the quality of VaR predictions.

All in all, our findings pinpoint that the SEP-POT model constitutes a reasonable promising alternative for forecasting extreme quantiles of financial returns and the daily VaR, especially for very small coverage rates. Undoubtedly, further examination of the theoretical properties of the SEP-POT model and its forecasting accuracy is needed. The model should be backtested using other classes of financial instruments and compared against other extreme risk models. However, there is a plethora of VaR models in the literature—therefore, there are no two or three candidate specifications against which the SEP-POT model should be benchmarked and compared. Only among the point process-based POT models there have been variants put forward, including the ACD-POT model (which is based on the dynamic specifications of time, i.e., duration, that elapses between consecutive extreme losses [6,7,8]) or the ACI-POT model (with its multivariate extensions) that provides an explicit autoregressive specification for the intensity function [13]. All these dynamic versions of POT models exploit both strands of the literature: the point process theory and the EVT, accounting for the clustering of extreme losses and the heavy-tailness of the loss distribution. The SEP-POT model is also suitable tailored to these features but also explicitly accounts for the discreteness of times between extreme losses. The empirical findings in this paper provide much support for our SEP-POT model. However, further efforts should be focused on benchmarking and comparison with a broader range of methods under the same settings (i.e., the same data and the same period).

## 4. Conclusions

We proposed a new self-exciting probability POT model for forecasting the risk of extreme losses. Existing methods within the point process approach to POT models pursue a continuous-time framework and therefore involve specification of an intensity function. Our model is inspired by leading research in this area but is based on observation of the real-world data as we built our model for discrete time. Hence, our model is a dynamic version of a POT model where extreme losses might occur upon a sequence of indivisible time units (i.e., days). Instead of delivering a new functional form for a conditional intensity of the point process, we propose its natural discrete counterpart being the conditional probability of experiencing an extreme event on a given day. This conditional probability is described in a dynamic fashion, allowing the recent events to have a greater effect than the distant ones. Thus, extreme losses arrive according to a self-exciting process, which allows for a realistic capturing of their clustering properties. The functional form of the conditional probability in the SEP-POT model resembles the conditional intensity function used in ETAS models. However, we rely on discrete weighting functions based on at-zero-truncated negative binomial (NegBin) distribution to provide a weight for the influence of past events.

Our move toward the discrete-time setup is backed up by the descriptive analysis of the data. On average, the probability mass for nearly 45% of the time intervals between extreme-loss days is distributed upon a set of discrete values ranging from one up to five days, and the shortest one-day-long duration has a relative frequency of 12% (for the threshold *u* set equal to the 95%-quantile of the unconditional distribution for negated returns). Accordingly, the motivation of the SEP-POT model lies in allowing the data to speak for itself. Using the at-zero-truncated NegBin distribution as a weighting function in the equation for the conditional probability of extreme loss, we try to tailor the method to the data specificity. The conditional distribution for the magnitudes of threshold exceedances also remain in line with this approach. We specify the evolution of the threshold exceedance magnitudes in a self-exciting fashion utilizing the weighting scheme based on the geometric probability density function. Accordingly, the sizes of more distant threshold exceedances have less effect on the current magnitudes of extreme losses than the more recent events do.

The backtesting results stay in favour of the SEP-POT VaR model. We used four backtesting procedures to check the practical utility of our approach for seven major stock indexes and three currency pairs both in- and out-of-sample. The out-of-sample period covered as much as over five years involving the series of catastrophic downswings in equity prices due to the COVID-19 pandemic in March 2020. We compared VaR forecasts delivered by the SEP-POT model with three widely recognized alternatives: self-exciting intensity (Hawkes) POT-VaR, skewed-t-GARCH VaR and Gaussian GARCH VaR model. Outcomes of backtesting procedures pinpoint that the SEP-POT model for VaR is a good alternative to existing methods.

The standard structure of the SEP-POT model offers several interesting generalizations. For example, it is possible to explain the conditional probability of an extreme loss with some covariates. Some potential candidate explanatory variables include price volatility measures such as high-low price ranges and measures of realized volatility. For stock indexes, some valuable information can be found in volatility indexes such as the CBOE volatility (VIX) index for the U.S. equity market. Unlike existing point process-based POT models, the merits of the SEP-POT model seem to lie in its discrete-time nature. Indeed, the Bernoulli log-likelihood function given in Equation (18) makes it easy to update an information set in the SEP-POT model on a regular, day-by-day basis. Another interesting generalization of the SEP-POT model could be to add the multi-excitation effect caused by different types of events. For example, the conditional probability of an extreme loss on one market could be additionally co-triggered by crashes observed in another market. Finally, the contemporaneous spillover effect between different markets can be captured using multivariate extensions of the SEP-POT model, for example based on extreme copula functions. These issues are left for further research.

## Figures and Tables

**Table 1:** Descriptive statistics for the threshold exceedance durations and the threshold exceedance magnitudes for the CAC 40, DAX 30, and FTSE 100 indexes. (Q(k) denotes the Ljung-Box test statistics for the lack of autocorrelation up to *k*-th order; Q(k) ***, Q(k) **, and Q(k) * denote the statistics significant at the 1%, 5%, and 10% levels).

	CAC 40		DAX 30		FTSE 100	
	**In-Sample**	**Out-of-Sample**	**In-Sample**	**Out-of-Sample**	**In-Sample**	**Out-of-Sample**
no. of						
daily returns	8574	1342	8563	1326	7826	1327
threshold value	0.021	0.021	0.021	0.021	0.017	0.017
no. of						
exceedances (*n*)	429	48	428	59	391	53
	threshold exceedance durations					
Min	1	1	1	1	1	1
Max	397	414	378	345	304	205
$\frac{\#1_{\left\{t_{i}-t_{i-1}=1\right\}}}{n}$	0.121	0.104	0.135	0.119	0.128	0.113
$\frac{\#1_{\left\{t_{i}-t_{i-1}=2\right\}}}{n}$	0.128	0.104	0.138	0.085	0.110	0.132
$\frac{\#1_{\left\{t_{i}-t_{i-1}=3\right\}}}{n}$	0.086	0.145	0.096	0.153	0.087	0.132
$\frac{\#1_{\left\{t_{i}-t_{i-1}<=5\right\}}}{n}$	0.441	0.438	0.486	0.424	0.455	0.453
$\frac{\#1_{\left\{t_{i}-t_{i-1}<=10\right\}}}{n}$	0.629	0.625	0.645	0.610	0.652	0.566
Mean	19.965	27.917	19.988	22.441	19.992	25
SD	40.002	66.572	41.786	49.212	40.245	42.287
Q(5)	83.307 ***	10.304 *	101.787 ***	5.688	69.617 ***	15.757 ***
Q(10)	91.822 ***	11.844	125.821 ***	9.206	108.4010 ***	20.790 **
Q(15)	95.692 ***	15.182	131.185 ***	11.796	134.1770 ***	31.453 ***
	threshold exceedance magnitudes					
Min	<0.001	<0.001	<0.001	<0.001	<0.001	<0.001
Max	0.130	0.110	0.116	0.109	0.114	0.099
Mean	0.011	0.015	0.012	0.012	0.010	0.012
SD	0.014	0.020	0.014	0.017	0.012	0.016
Q(5)	92.293 ***	14.309 **	51.668 ***	16.47251 ***	174.289 ***	27.924 ***
Q(10)	95.931 ***	16.093 *	61.467 ***	17.225 *	198.963 ***	28.375 ***
Q(15)	97.429 ***	17.579	66.683 ***	18.845	200.149 ***	28.954 **

**Table 2:** Maximum likelihood (ML) parameter estimates of the self-exciting probability peaks-over-threshold (SEP-POT) for the CAC 40 and DAX 30 indices. Standard errors given in brackets.

Parameter	CAC 40		DAX 30		FTSE 100	
model for the probability of threshold exceedances						
μ	0.017	(0.002)	0.014	(0.002)	0.012	(0.002)
α	0.710	(0.052)	0.776	(0.053)	0.823	(0.063)
ω	13.452	(1.583)	13.919	(1.531)	20.923	(4.831)
κ	0.719	(0.086)	0.751	(2.806)	1.655	(0.490)
model for the sizes of threshold exceedances						
$\mu_{s}$	0.006	(0.000)	0.006	(0.001)	0.005	(0.001)
$\alpha_{s}$	2.225	(0.389)	2.242	(0.388)	2.583	(0.457)
$\omega_{s}$	7.161	(2.272)	10.439	(3.793)	12.624	(4.075)
ξ	0.122	(0.044)	0.110	(0.042)	0.070	(0.038)
AIC	16.001		15.997		15.978	
BIC	72.454		72.439		71.701	

## References

[1] Jorion P. (2006). Value at Risk: The New Benchmark for Managing Financial Risk.

[2] Christoffersen P.F. (2012). Elements of Financial Risk Management.

[3] McNeil A., Frey R. (2000). Estimation of tail-related risk measures for heteroscedastic financial time series: An extreme value approach. J. Empir. Financ..

[4] Chavez-Demoulin V., Davidson A.C., McGill J.A. (2005). Estimating value-at-risk: A point process approach. Quant. Financ..

[5] Chavez-Demoulin V., McGill J.A. (2012). High-frequency financial data modeling using Hawkes processes. J. Bank. Financ..

[6] Hamidieh K., Stoev S., Michailidis G. (2013). Intensity-based estimation of extreme loss event probability and value at risk. Appl. Stoch Model Bus..

[7] Herrera R., Schipp B. (2013). Value at risk forecasts by extreme value models in a conditional duration framework. J. Empir. Financ..

[8] Pyrlik V. (2013). Autoregressive conditional duration as a model for financial market crashes prediction. Physica A.

[9] Herrera R., González N. (2014). The modeling and forecasting of extreme events in electricity spot markets. Int. J. Forecast..

[10] Grothe O., Korniichuk V., Manner H. (2014). Modeling multivariate extreme events using self-exciting point processes. J. Economet..

[11] Clements A.E., Herrera R., Hurn A.S. (2015). Modelling interregional links in electricity price spikes. Energy Econ..

[12] Herrera R., Clements A.E. (2018). Point process models for extreme returns: Harnessing implied volatility. J. Bank. Financ..

[13] Hautsch N., Herrera R. (2020). Multivariate dynamic intensity peaks-over-threshold models. J. Appl. Econ..

[14] Stindl T., Chen F. (2019). Modeling extreme negative returns using marked renewal Hawkes processes. Extremes.

[15] Hautsch N. (2012). Econometrics of Financial High-Frequency Data.

[16] Engle R.F., Russell J.R. (1998). Autoregressive conditional duration: A new model for irregularly spaced transaction data. Econometrica.

[17] Pacurar M. (2008). Autoregressive conditional duration (ACD) models in finance: A survey of the theoretical and empirical literature. J. Econ. Surv..

[18] Porter M.D., White G. (2012). Self-exciting hurdle models for terrorist activity. Ann. Appl. Stat..

[19] Daley D.J., Vere-Jones D. (2003). An Introduction to the Theory of Point Processes, Volume I: Elementary Theory and Methods.

[20] McNeil A.J., Frey R., Embrechts P. (2005). Quantitative Risk Management: Concepts, Techniques and Tools.

[21] Lewis P.A.W., Shedler G.S. (1976). Statistical Analysis of Non-Stationary Series of Events in a Data Base System.

[22] Liesenfeld R., Nolte I., Pohlmeier W. (2006). Modelling Financial Transaction Price Movements: A Dynamic Integer Count Data Model. Appl. Econ..

[23] Bień K., Nolte I., Pohlmeier W. (2011). An inflated multivariate integer count hurdle model: An application to bid and ask quote dynamics. J. Appl. Economet..

[24] Yamai Y., Yoshiba T. (2002). Comparative analyses of expected shortfall and value-at-risk: Their validity under market stress. Monet. Econ. Stud..

[25] Kupiec P.H. (1995). Techniques for Verifying the Accuracy of Risk Measurement Models. J. Deriv..

[26] Christoffersen P.F. (1998). Evaluating Interval Rorecasts. Int. Econ. Rev..

[27] Engle R.F., Manganelli S. (2004). CAViaR: Conditional Autoregressive Value at Risk by Regression Quantiles. J. Bus. Econ. Stat..

[28] Dumitrescu E., Hurlin C., Pham V. (2012). Backtesting Value-at-Risk: From Dynamic Quantile to Dynamic Binary Tests. Finance.

[29] Bank of International Settlements (2016). Triennial Central Bank Survey. Foreign Exchange Turnover in April 2016.

[30] Fernandes M., Grammig J. (2005). Non-parametric specification tests for conditional duration models. J. Economet..

[31] Basel Committee on Banking Supervision (2015). Minimum Capital Requirements for Market Risk.

[32] Roncalli T. (2020). Handbook of Financial Risk Management.

